# Supplementary material for: Heating a dipolar quantum fluid into a solid
Source: Nat Commun. 2023 Apr 4;14:1868. doi: 10.1038/s41467-023-37207-3 (PMC10073146; doi:10.1038/s41467-023-37207-3)
Supplement: Supplementary file 1 — Supplementary Information [file 41467_2023_37207_MOESM1_ESM.pdf]

Supplementary Information
Heating a dipolar quantum fluid into a solid

J. Sánchez-Baena,[1,2] Claudia Politi,[3,4] F. Maucher,[5,1] Francesca Ferlaino,[3,4] and T. Pohl[1]

[1] *Center for Complex Quantum Systems, Department of Physics and Astronomy, Aarhus University, DK-8000 Aarhus C, Denmark*

[2] *Departament de Física, Universitat Politècnica de Catalunya, Campus Nord B4-B5, 08034 Barcelona, Spain*

[3] *Institut für Quantenoptik und Quanteninformation, Österreichische Akademie der Wissenschaften, Innsbruck, Austria*

[4] *Institut für Experimentalphysik, Universität Innsbruck, Austria*

[5] *Departament de Física, Universitat de les Illes Balears & IAC-3, Campus UIB, E-07122 Palma de Mallorca, Spain*

SUPPLEMENTARY DISCUSSIONS

Supplementary Discussion1: Thermodynamics of dipolar Bose Einstein condensates

Here, we present a more detailed discussion of the thermodynamics of weakly interacting dipolar Bose Einstein condensates (BECs), and describe the derivation of the finite-temperature extended Gross-Pitaevskii equation (TeGPE) based on Bogoliubov theory and the local density approximation (LDA). To this end, we start from the thermodynamic properties of a homogenous infinitely extended system with constant BEC density ρ, which is eventually identified by the local density $|\psi(\mathbf{r})|^2 = \rho$ of the condensate.

The grand canonical potential of the homogenous BEC at finite temperature T is defined as

$$\Omega = E - TS - \mu N \ , \tag{1}$$

in terms of the internal energy E, the entropy S and the particle number N. In the limit of weak interactions, one can use Bogoliubov theory to obtain to leading order in the fluctuations [1]

$$\Omega = E_0 + \frac{V}{\beta}\int \frac{d\mathbf{k}}{(2\pi)^3} \ln\left(1 - e^{-\beta\varepsilon_{\mathbf{k}}}\right) \ , \tag{2}$$

in terms of the grand canonical zero-temperature energy $E_0 = E_{\rm MF} + E_{\rm LHY} - \mu N$ and the spectrum $\varepsilon_{\mathbf{k}} = \sqrt{\tau_{\mathbf{k}}\left(\tau_{\mathbf{k}} + 2\rho\tilde{V}(\mathbf{k})\right)}$ of Bogoliubov excitations, where $\beta = 1/k_B T$, $\tau_{\mathbf{k}} = \frac{\hbar^2 k^2}{2m}$, and V is the volume of the system. The energy of the zero-temperature ground state is composed of the mean field energy $E_{\rm MF}$ and the Lee-Huang-Yang correction $E_{\rm LHY}$, which accounts for quantum fluctuations. The Fourier transform of the interaction potential is given by [2]

$$\tilde{V}(\mathbf{k}) = \frac{4\pi\hbar^2 a}{m} + \frac{4\pi\hbar^2 a_{\rm d}}{m}\left(3\frac{k_y^2}{k^2} - 1\right) \ . \tag{3}$$

Let us now compute the grand canonical potential for an inhomogeneous systems within the LDA. This is done by computing the energy density Ω/V for the homogenous system, replacing the condensate density $\rho = N_c/V \to |\psi(\mathbf{r})|^2$ and integrating over $\mathbf{r}$. This yields for the mean field energy

$$E_{\rm MF} = \int d\mathbf{r}\left\{-\psi^*(\mathbf{r})\frac{\hbar^2}{2m}\nabla^2\psi(\mathbf{r}) + \frac{g}{2}|\psi(\mathbf{r})|^4 + U(\mathbf{r})|\psi(\mathbf{r})|^2 + \frac{1}{2}\int d\mathbf{r}' V_{\rm dd}(\mathbf{r}-\mathbf{r}')|\psi(\mathbf{r}')|^2|\psi(\mathbf{r})|^2\right\}. \tag{4}$$

The LHY correction to the ground state energy is given by [3]

$$E_{\rm LHY} = \int d\mathbf{r}\ E_{\rm LHY}/V\Big|_{\rho=|\psi(\mathbf{r})|^2} = \int d\mathbf{r}\ \frac{64}{15\sqrt{\pi}} g\sqrt{a^3}\,{\rm Re}\{Q_5(\epsilon_{\rm dd})\}|\psi(\mathbf{r})|^5 + \mathcal{O}(\delta N^2), \tag{5}$$

where Re{} denotes the real part and [4]

$$Q_5(\epsilon_{\rm dd}) = \int_0^1 du\left(1 - \epsilon_{\rm dd} + 3\epsilon_{\rm dd}u^2\right)^{5/2} \ , \tag{6}$$

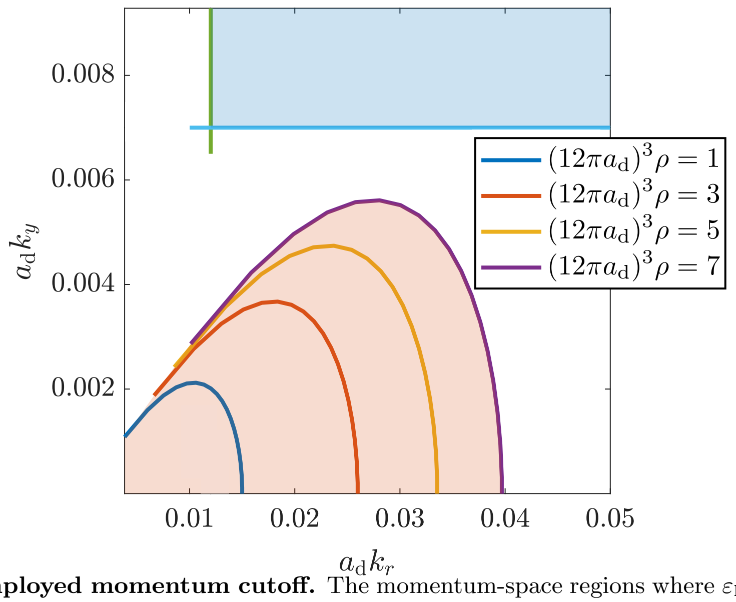

Fig. S1. **Illustration of employed momentum cutoff.** The momentum-space regions where $\varepsilon_{\mathbf{k}}$ turns imaginary are marked by the red shaded areas and by the solid lines for different densities as indicated in the figure. The straight lines show the momentum cutoff $k_r^{(c)}a_{\mathrm{d}} = 0.012$ (vertical green line) and $k_y^{(c)}a_{\mathrm{d}} = 0.007$ (horizontal blue line) as used in the calculations for Figs.1(b) and 2 of the main text. The blue shaded area indicates the corresponding integration region as used to determine H_{th} according to Eq.(21).

with $\epsilon_{\mathrm{dd}} = a_{\mathrm{d}}/a$ and $g = 4\pi\hbar^2 a/m$. The temperature-dependent term takes the form

$$F_{\mathrm{T}} = \int d\mathbf{r} \left\{ \int \frac{d\mathbf{k}}{(2\pi)^3\beta} \ln\left(1 - e^{-\beta\varepsilon_{\mathbf{k}}}\right)\Big|_{\rho=|\psi(\mathbf{r})|^2} \right\} + \mathcal{O}\big(\delta N^2\big) \ , \tag{7}$$

and

$$\mu N \simeq \mu \int d\mathbf{r}|\psi(\mathbf{r})|^2 \ , \tag{8}$$

The grand canonical potential $\Omega = E_{\mathrm{MF}} + E_{\mathrm{LHY}} + F_{\mathrm{T}} - \mu N$ is minimal in equilibrium. We can thus minimize Ω,

$$\frac{\delta\Omega}{\delta\psi^*} = 0, \tag{9}$$

to obtain the condensate wave function $\psi(\mathbf{r})$ in thermal equilibrium. The required functional derivatives are readily obtained from Eqs. (4)-(8)

$$\frac{\delta E_{\mathrm{MF}}}{\delta\psi^*} = -\frac{\hbar^2}{2m}\nabla^2\psi(\mathbf{r}) + g|\psi(\mathbf{r})|^2\psi(\mathbf{r}) + U(\mathbf{r})\psi(\mathbf{r}) + \int d\mathbf{r}' V_{\mathrm{dd}}(\mathbf{r}-\mathbf{r}')|\psi(\mathbf{r}')|^2\psi(\mathbf{r}) \tag{10}$$

$$\frac{\delta E_{\mathrm{LHY}}}{\delta\psi^*} = \frac{32}{3\sqrt{\pi}} g\sqrt{a^3} Q_5(\epsilon_{\mathrm{dd}})|\psi(\mathbf{r})|^3\psi(\mathbf{r}) \tag{11}$$

$$\frac{\delta F_{\mathrm{T}}}{\delta\psi^*} = \psi(\mathbf{r})\ \frac{\partial}{\partial\rho}\int \frac{d\mathbf{k}}{(2\pi)^3\beta} \ln\left(1 - e^{-\beta\varepsilon_{\mathbf{k}}}\right)\Big|_{\rho=|\psi(\mathbf{r})|^2} = \psi(\mathbf{r}) \int \frac{d\mathbf{k}}{(2\pi)^3} \frac{1}{(e^{\beta\varepsilon_{\mathbf{k}}}-1)} \tilde{V}(\mathbf{k}) \frac{\tau_{\mathbf{k}}}{\varepsilon_{\mathbf{k}}}\Big|_{\rho=|\psi(\mathbf{r})|^2} \tag{12}$$

$$\frac{\delta \mu N}{\delta\psi^*} = \mu\psi(\mathbf{r}) \ . \tag{13}$$

Using

$$|u(\mathbf{k})|^2 = \frac{1}{2}\left(\frac{\tau_{\mathbf{k}} + \rho\tilde{V}(\mathbf{k})}{\varepsilon_{\mathbf{k}}} + 1\right) \tag{14}$$

$$|v(\mathbf{k})|^2 = \frac{1}{2}\left(\frac{\tau_{\mathbf{k}} + \rho\tilde{V}(\mathbf{k})}{\varepsilon_{\mathbf{k}}} - 1\right) \tag{15}$$

$$u(\mathbf{k})v^*(\mathbf{k}) = \rho\tilde{V}(\mathbf{k})/2\varepsilon_{\mathbf{k}} \tag{16}$$

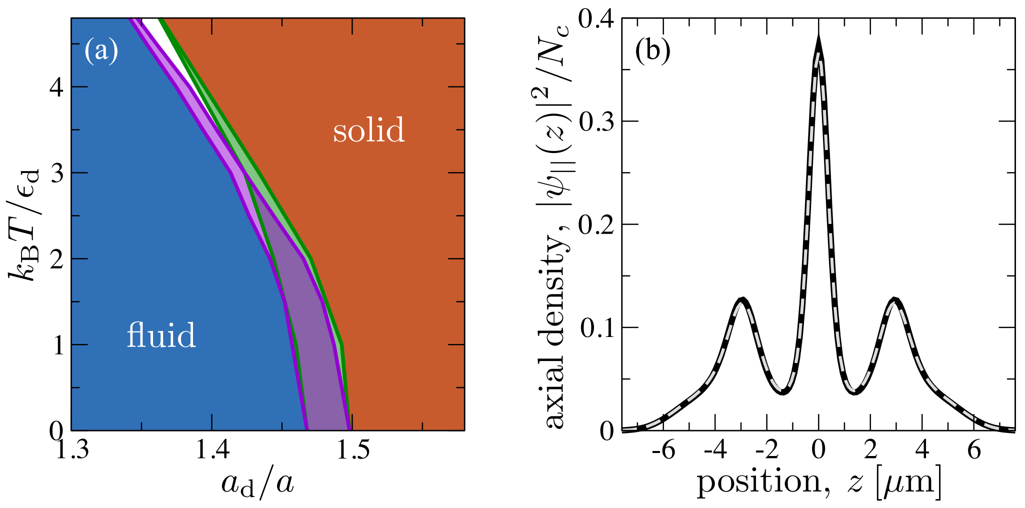

Fig. S2. **Influence of the momentum cutoff.** (a) Effect of the low-momentum cutoff on the thermodynamic phase diagram. The purple-shaded area corresponds to the coexistence region shown in Fig. 1(a) of the main text, for which the cut-offs $k_y^{(c)} a_{\rm d} = 0.007$ and $k_r^{(c)} a_{\rm d} = 0.012$ have been used. The green-shaded area shows the coexistence region obtained for larger values of $k_y^{(c)} a_{\rm d}$ and $k_r^{(c)} a_{\rm d}$, increased by 30%. While we observe small deviations with increasing temperature, the low-temperature phase boundaries are found to be largely insensitive to the precise choice of the momentum cutoff. In panel (b) we show the axial BEC density for the parameters of Fig.3 of the main text. The black solid line is obtain for cut-off values of $k_y^{(c)} a_{\rm d} = 0.007$ and $k_r^{(c)} a_{\rm d} = 0.012$ and corresponds to the theory curve shown in Fig.3 of the main text. The dashed grey line is obtained for 30% larger values of the momentum cutoff. Both results are virtually identical.

for the Bogoliubov amplitudes $u(\mathbf{k})$, $v(\mathbf{k})$, we can rewrite Eq. (12) as

$$\frac{\delta F_{\rm T}}{\delta\psi^*} = \psi(\mathbf{r}) \left\{ \int \frac{d\mathbf{k}}{(2\pi)^3} \frac{1}{(e^{\beta\varepsilon_{\mathbf{k}}} - 1)} \tilde{V}(\mathbf{k}) \left(|u(\mathbf{k})|^2 + |v(\mathbf{k})|^2 - 2u(\mathbf{k})v^*(\mathbf{k}) \right) \bigg|_{\rho=|\psi(\mathbf{r})|^2} \right\}. \tag{17}$$

By substituting Eqs. (10), (11), (13) and (17) into Eq. (9),

$$\mu\psi(\mathbf{r}) = \frac{\delta E_{\rm MF}}{\delta\psi^*} + \frac{\delta E_{\rm LHY}}{\delta\psi^*} + \frac{\delta F_{\rm T}}{\delta\psi^*}, \tag{18}$$

we finally obtain the TeGPE

$$\mu\psi(\mathbf{r}) = \left(-\frac{\hbar^2\nabla^2}{2m} + U(\mathbf{r}) + \frac{4\pi\hbar^2 a}{m}|\psi(\mathbf{r})|^2 + \int d\mathbf{r}' V_{\rm dd}(\mathbf{r}-\mathbf{r}')|\psi(\mathbf{r}')|^2 + H_{\rm qu}(\mathbf{r}) + H_{\rm th}(\mathbf{r}) \right) \psi(\mathbf{r}), \tag{19}$$

where

$$\varepsilon_{\rm d}^{-1} H_{\rm qu}(\mathbf{r}) = \frac{96(4\pi)^3}{\sqrt{\pi}} \frac{a_{\rm d}^2}{a^2} Q_5(\epsilon_{\rm dd}) \left| a^{3/2}\psi(\mathbf{r}) \right|^3, \tag{20}$$

and

$$\begin{aligned} \varepsilon_{\rm d}^{-1} H_{\rm th}(\mathbf{r}) &= \int \frac{d\mathbf{k}}{(2\pi)^3} \frac{|u(\mathbf{k})|^2 + |v(\mathbf{k})|^2 - 2u(\mathbf{k})v^*(\mathbf{k})}{e^{\beta\varepsilon_{\mathbf{k}}} - 1} \frac{\tilde{V}(\mathbf{k})}{\varepsilon_{\rm d}} \\ &= \int \frac{d\mathbf{k}}{(2\pi)^3} \frac{1}{e^{\beta\varepsilon_{\mathbf{k}}} - 1} \frac{\tilde{V}(\mathbf{k})}{\varepsilon_{\rm d}} \frac{\tau_{\mathbf{k}}}{\varepsilon_{\mathbf{k}}}\ . \end{aligned} \tag{21}$$

The first line in Eq.(21) illustrates directly the role of interactions between the condensate and thermal Bogoliubov excitations in generating the focussing nonlinearity $H_{\rm th}(\mathbf{r})$ in the wave equation.

As mentioned in the main text, the LDA entails a long-wavelength divergence when evaluating the potential term $H_{\rm th}$. Due to the cylindrical symmetry of the interaction potential Eq.(3), we can reexpress the integration in Eq.(21) solely in terms of k_y and $k_r = \sqrt{k_x^2 + k_z^2}$.

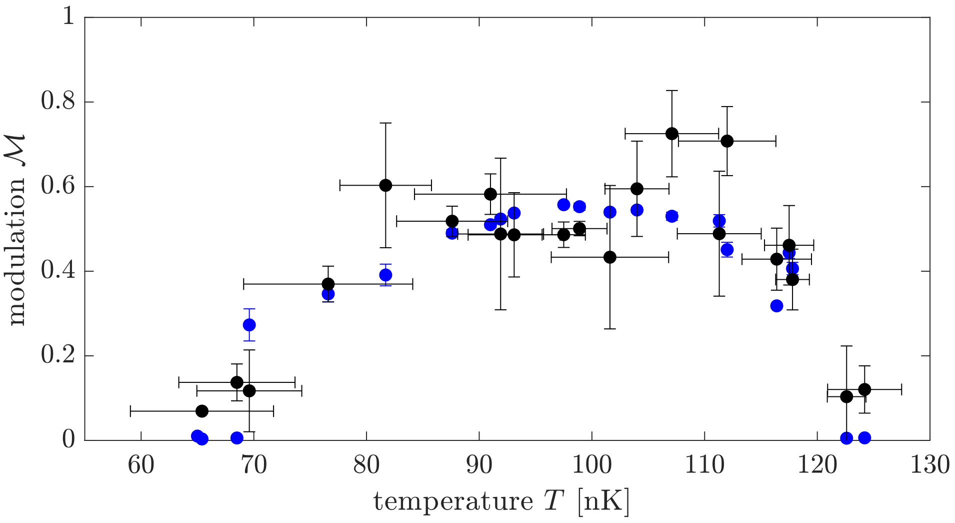

Fig. S3. **Density-modulation vs. temperature.** Calculated (blue dots) and measured (black dots) values of the modulation contrast $\mathcal{M}$ as a function of temperature. The different temperatures are obtained during the evaporative cooling cycle, such that the atom number varies as well. The experimental error bars indicate the standard deviation of the temperature measurements as well as the statistical uncertainty of the measured contrast, obtained from the standard deviation of the different density-profile images from repeated measurements. The vertical error bars of the theoretical results reflect the experimental uncertainty of the number of condensate atoms for a given temperature and is obtained from additional simulations for the upper and lower estimate of the density within the experimental uncertainty.

The divergence arises when $a < a_{\rm d}$, i.e. when the homogenous condensate would become unstable in the absence of quantum fluctuations and undergo dipolar collapse. Formally, the Fourier transform of the interaction potential, Eq. (3), becomes negative for some values of $\mathbf{k}$, which can lead to imaginary excitation energies $\varepsilon_{\mathbf{k}}$. This defines a region in $\mathbf{k}$-space where the condensate would become unstable and which causes a divergence of the integral in Eq.(21) at low momenta. In finite systems, however, such long wavelength excitations are not supported and one can introduce a low-momentum cutoff. For a given characteristic size of the condensate L_x, L_y, and L_z along each cartesian axis, we can, thus, use $k_y^{(c)} = 2\pi/L_y$ and $k_r^{(c)} = \sqrt{(2\pi/L_x)^2 + (2\pi/L_z)^2}$ as low-momentum cutoffs in the integral in Eq.(21).

For the calculations of Figs.1(a) and 2, the condensate has a typical size of $L_x = 527a_{\rm d}$, $L_y = 905a_{\rm d}$, and $L_z \to \infty$, while the simulations for Figs.1(b) and 3 give a typical condensate size of $L_x = 3.65\mu$m, $L_y = 6.25\mu$m, and $L_z = 13\mu$m. The precise values vary only slightly for the different parameters depicted in the figures. Using these values we obtain characteristic momentum cutoffs of $k_y^{(c)}a_{\rm d} = 0.007$ and $k_r^{(c)}a_{\rm d} = 0.012$ for the infinitely elongated condensate (Figs.1(a) and 2) and $k_y^{(c)} = 1\mu\text{m}^{-1}$ and $k_r^{(c)} = 1.8\mu\text{m}^{-1}$ for the fully confined BEC considered in Figs.1(b) and 3.

Figure S1 shows the momentum-space region in which the $\varepsilon_{\mathbf{k}}$ becomes imaginary along with the integration region determined by the momentum cutoff for the infinitely elongated BEC. In order to examine the significance of the momentum cutoff, we recalculated the phase diagram shown in Fig.1(a) of the main text with a larger momentum cutoff, increased by 30%. Both results are compared in Fig. S2(a). Since the Bose distribution $f_{\mathbf{k}} = 1/(e^{\beta\varepsilon_{\mathbf{k}}} - 1)$ features growing populations of low-energies modes with longer wavelengths with increasing temperature, we expectedly observe quantitative deviations at higher temperatures. At lower temperatures, $k_{\rm b}T \lesssim 2\epsilon_{\rm d}$, however, the two calculations yield nearly identical results despite the significant change of the momentum cutoff. More importantly, the observed effect of a temperature-driven supersolid formation is robust with respect to the precise value of the momentum cutoff. The predictive power of the simulations is further demonstrated in Fig. S2(b), where we show the calculated density profile of Fig.3 of the main text and find nearly identical results for the two different momentum cutoffs used in the simulations.

Supplementary Discussion2: Modulation contrast of the condensate density in theory and experiment

In order to construct the phase diagram shown in Fig.1b of the main text, we have determined the modulation contrast $\mathcal{M}$ from our calculated condensate densities and measured density profiles, as described in the Methods section. Figure. S3 demonstrates that we not only obtain good agreement for the location of the fluid-solid transition

(cf. Fig.1(b) of the main text) but also obtain good quantitative agreement for the modulation contrast for all densities and temperatures that are realized during the evaporative cooling process of the gas.

The experimental density profiles $\mathcal{M} \lesssim 0.2$ do not exhibit a clear density modulation, and the finite value of $\mathcal{M}$ stems from background noise. We have therefore used a critical value of 0.2 to distinguish between modulated and unmodulated states and generate the coloring of the experimental data points in Fig.1(b) of the main text.

SUPPLEMENTARY REFERENCES

[1] Christopher Gaul and Cord Müller, "A grand-canonical approach to the disordered bose gas," Applied Physics B **117** (2014), 10.1007/s00340-014-5805-2.

[2] P Blair Blakie, D Baillie, and Sukla Pal, "Variational theory for the ground state and collective excitations of an elongated dipolar condensate," Communications in Theoretical Physics **72**, 085501 (2020).

[3] A. R. P. Lima and A. Pelster, "Beyond mean-field low-lying excitations of dipolar bose gases," Phys. Rev. A **86**, 063609 (2012).

[4] Aristeu R. P. Lima and Axel Pelster, "Quantum fluctuations in dipolar bose gases," Phys. Rev. A **84**, 041604 (2011).
